# Specific Multilocus Variable-Number Tandem-Repeat Analysis Genotypes of *Mycoplasma pneumoniae* Are Associated with Diseases Severity and Macrolide Susceptibility

**DOI:** 10.1371/journal.pone.0082174

**Published:** 2013-12-18

**Authors:** Jiuxin Qu, Xiaomin Yu, Yingmei Liu, Yudong Yin, Li Gu, Bin Cao, Chen Wang

**Affiliations:** 1 Department of Infectious Diseases and Clinical Microbiology, Beijing Chaoyang Hospital, Beijing Institute of Respiratory Medicine, Capital Medical University, Beijing, China; 2 Beijing Institute of Respiratory Medicine, Beijing Key Laboratory of Respiratory and Pulmonary Circulation Disorders, Beijing Hospital, China Ministry of Health, Beijing; Miami University, United States of America

## Abstract

Clinical relevance of multilocus variable-number tandem-repeat (VNTR) analysis (MLVA) in patients with community-acquired pneumonia (CAP) by *Mycoplasma pneumoniae* (*M. pneumoniae*) is unknown. A multi-center, prospective study was conducted from November 2010 to April 2012. Nine hundred and fifty-four CAP patients were consecutively enrolled. *M. pneumoniae* clinical isolates were obtained from throat swabs. MLVA typing was applied to all isolates. Comparison of pneumonia severity index (PSI) and clinical features among patients infected with different MLVA types of *M. pneumoniae* were conducted. One hundred and thirty-six patients were positive with *M. pneumoniae* culture. The clinical isolates were clustered into 18 MLVA types. One hundred and fourteen (88.3%) isolates were resistant to macrolide, covering major MLVA types. The macrolide non-resistant rate of *M. pneumoniae* isolates with Mpn13-14-15-16 profile of 3-5-6-2 was significantly higher than that of other types (p≤0.001). Patients infected with types U (5-4-5-7-2) and J (3-4-5-7-2) had significantly higher PSI scores (p<0.001) and longer total duration of cough (p = 0.011). Therefore it seems that there is a correlation between certain MLVA types and clinical severity of disease and the presence of macrolide resistance.

## Introduction

*Mycoplasma pneumoniae* (*M. pneumoniae*) is a common and important pathogen of community-acquired pneumonia (CAP), accounting for 10%–30% of cases [1]–[4]. Genotyping of clinical isolates is very important for clinical and epidemiological study of *M. pneumoniae* infections. However, isolates are not well differentiated using the most common typing methods based on the analysis of the gene encoding the P1 protein [5], [6]. In 2009, Dégrange *et al* have established the multilocus variable-number tandem-repeat (VNTR) analysis (MLVA) method based on 5 VNTRs as Mpn1-13-14-15-16, and clustered 265 *M. pneumoniae* clinical isolates into 26 MLVA types [7]. In 2011, the same group has successfully confirmed a single-clone spread of type J (3-4-5-7-2) *M. pneumoniae* among children in a French primary school and their household contacts. Although the VNTR locus Mpn1 is reported to be unstable in both clinical isolates and in laboratory passages, MLVA genotyping of *M. pneumoniae* still exceeds the discriminatory power of previous typing methods based on P1 gene [8], [9].

Macrolide-resistant *M. pneumoniae* have been spreading for 10 years worldwide, with prevalences ranging from below 10% in Europe, approximately 30% in Israel [10] and ranging from 69% to 92% in Asia [2], [11], [12]. One report found there was a possible association between the MLVA type Z (7-4-5-7-2) and macrolide resistance [13], whereas other reports found no association between macrolide resistance and MLVA types [9], [12], [14]. The evidence of clinical association between MLVA types and macrolide susceptibility is limited. More important, since almost all of the published reports focused on MLVA genotyping of *M. pneumoniae* isolates and method improvement [8], [9], [12], [15]–[17], the clinical relevance of MLVA types, such as the association with disease severity and clinical features, is still lacking.

In this multi-center and prospective study, we used MLVA typing method to analyze the *M. pneumoniae* clinical isolates, and tried to investigate the possible associations between MLVA genotypes and clinical features, including macrolide susceptibility and diseases severity.

## Materials and Methods

### Study setting and design

A prospective study was conducted in 12 general hospitals in Beijing, as described in former report [18]. Between November 2010 and April 2012, patients (aged 14 years or above) who came to the hospitals and met the inclusion criteria of CAP [19] were enrolled. Patients with HIV infection; neutropenia or chemotherapy; pregnant; known or suspected active tuberculosis, no informed consents or specimens were excluded. Adolescents were defined as aged from 14 to 17 years old, and adults were ≥18 years old. The study was approved by Institutional Review Board of Beijing Chao-Yang Hospital (project approval number 10-KE-49). Written informed consents were provided by all adults and the parents of patients aged less than 18 years.

The following data were recorded on enrollment: age, gender, smoking status, comorbidities and antimicrobial treatment prior to enrollment, clinical symptoms, etc. All surviving patients were followed-up by telephone within 7 to 28 days after discharge, symptoms and signs were recorded daily. Pneumonia severity index (PSI) scores were calculated [20].

### *M. pneumoniae* Culture and Antimicrobial Susceptibility Test

Culture of *M. pneumoniae* was performed as described previously by Waites [21]. One hundred and thirty-six *M. pneumoniae* isolates were obtained from throat swabs (each specimen collected from one patient). All the isolates were identified by colony morphology and PCR assay.

Minimum inhibitory concentrations (MICs) were determined by broth microdilution methods with SP4 broth (Remel) [21]. Susceptibility tests were performed in triplicate. *M. pneumoniae* reference strain MPFH (ATCC 15531) were also included. According to CLSI guideline (M43-A, 31(19), 2011) for antimicrobial susceptibility for human mycoplasmas including *M. pneumoniae*, susceptible strains have a MIC of erythromycin and azithromycin ≤0.5 µg/ml and resistant strains have a MIC ≥1 µg/ml. The total length of the 23S ribosomal RNA (rRNA) gene of each *M. pneumoniae* isolate was amplified and sequenced by the method described previously [22]. The mutations of 23S rRNA have been determined by alignment with sequence of MPFH (ATCC 15531).

### MLVA and P1 Gene Typing

MLVA typing on *M. pneumoniae* isolates was performed using PCRs with fluorescently labeled primers targeting five VNTR loci, followed by capillary electrophoresis [7]. One reference strain of MPFH (ATCC 15531), was included as the positive control with MLVA type T (5-3-6-6-2). There were two peaks of molecular weight signal in Mpn1 of four samples, Mpn13 of one sample and Mpn15 of two samples. The numbers of VNTR were finally determined by sequencing of PCR products. P1 gene typing on *M. pneumoniae* isolates was performed using a multiplex PCR as described by Kenri [23].

### Statistical Analysis

Continuous variables were presented as means (± SD), or medians (with IQR) where appropriate. For categorical variables, the percentages were calculated. The PSI scores of patients infected with different MLVA types were compared using One-Way ANOVA and Tukey's pairwise comparison test. Comparisons of macrolide resistant rates with different VNTRs profiles were conducted using a Chi-square test, Bonferroni method was used for multiple comparisons with adjusted significance level as α = 0.05/3 = 0.0167. Comparisons of clinical features were conducted between MLVA type groups, using an Independent-Sample t-test or the Mann–Whitney test (SPSS for Windows 13.0).

## Results

### Patient Characteristics

From November 2010 to April 2012, a total of 954 CAP patients (aged 14–94 years) were enrolled, with 136 patients (aged 14–73 years) positive with *M. pneumoniae* culture, as shown in table 1. The comorbidities were mainly diabetes, heart diseases, cerebral vascular disease and COPD. Patients with *M. pneumoniae* infections were younger than those without (median age, 29 vs 47 years, respectively; p<0.001), less likely to be have underlying comorbidities (5.1%, 1.4%–8.8% vs 21.6%, 18.8%–24.4%, respectively; p<0.001) and smoking history (15.4%, 9.3%–21.5% vs 28.5%, 25.4%–31.6%, respectively; p = 0.002), had less severity (median PSI scores 30.0 vs 46.0, respectively; p<0.001). Cough was more common in *M. pneumoniae* pneumonia patients (97.8%, 95.3%–100.0% vs 90.7%, 88.7%–92.7%, respectively; p = 0.013).

**Table 1:** Comparison between patients with *M. pneumoniae* pneumonia and those without.

	*M. pneumoniae* group	Non *M. pneumoniae* group	p value
Patients number (n)	136	818	
Age (years)	29 (16)	47 (33)	<0.001
Adolescents, n (%)	19 (14.0, 8.2–19.8)	31 (3.8, 2.5–5.3)	<0.001
Male gender, n (%)	61 (44.9, 36.5–53.2)	479 (58.6, 55.2–62.0)	0.004
Comorbidities, n (%)	7 (5.1, 1.4–8.8)	177 (21.6, 18.8–24.4)	<0.001
Smoking, n (%)	21 (15.4, 9.3–21.5)	233 (28.5, 25.4–31.6)	0.002
Fever, n (%)	130 (95.6, 92.2–99.0)	748 (91.4, 89.5–93.3)	0.122
T_max_ (°C)	39.2 (0.8)	39.0 (1.0)	0.006
Cough, n (%)	133 (97.8, 95.3–100.0)	742 (90.7, 88.7–92.7)	0.006
Sputum, n (%)	104 (76.5, 69.4–83.6)	568 (69.4, 66.2–72.6)	0.105
PSI score	30.0 (22.0)	46.0 (38.0)	<0.001
Site of care, n (%)			0.079
Inpatients	80 (58.8, 50.5–67.1)	547 (66.9, 63.7–70.1)	
Ward	80 (58.8, 50.5–67.1)	541 (66.3, 63.1–69.5)	
ICU	0 (0.0)	6 (0.6, 0.7–1.1)	

Note:The data are presented as n (%, 95% confidence intervals) or median (IQR).

### Macrolide Susceptibility and MLVA Typing of Clinical Isolates

In vitro activities of nine antimicrobials are listed in Table S1. Among 136 isolates, 114 (83.8%) were resistant to macrolide with MIC ≥1 µg/mL to erythromycin. These erythromycin-resistant isolates also showed cross-resistance to clarithromycin and azithromycin. Results of 23S ribosomal RNA (rRNA) gene sequencing indicated that all macrolide-resistant isolates harbored an A2063G mutation, and no such mutation had been found in non-resistant isolates.

One hundred and thirty-six *M. pneumoniae* clinical isolates were divided into 18 MLVA types, of which 13 types were previously reported [7], and the other five types were putative new MLVA types (shown in table 2), (2-4-5-7-3), (3-3-5-7-2), (3-4-5-7-1), (6-4-4-7-2) and (6-4-5-6-2). The most prevalent MLVA types are U (5-4-5-7-2), X (6-4-5-7-2), P (4-4-5-7-2), J (3-4-5-7-2) and E (2-4-5-7-2). One hundred and fourteen macrolide-resistant ones were clustered into 15 MLVA types (table 2), covering all major types. The macrolide non-resistant isolates were mainly consisted of type M (8/9, 4-3-5-6-2), type S (5/7, 5-3-5-6-2) and type V (5/5, 6-3-5-6-2). Among all clinical isolates, 111 were p1 gene type I and 25 were type II (table 2). As shown in table 3, the macrolide non-resistant rate of *M. pneumoniae* with Mpn13-14-15-16 profile of 3-5-6-2, when compared with that of others, was significantly higher (with p value as <0.001 and 0.001).

**Table 2:** Results of MLVA and P1 gene types according to macrolide susceptibility and comparison of PSI scores among CAP patients.

MLVA type	Mpn1	Mpn13	Mpn14	Mpn15	Mpn16	Total	P1 gene type		ML^s^	ML^r^	PSI score^a^
							Type I	TypeII			
B	2	3	5	6	2	2	0	2	2	0	ND^b^
E	2	4	5	7	2	10	10	0	0	10	28.1±14.3
G	3	3	5	6	2	2	0	2	1	1	ND
J*	3	4	5	7	2	15	15	0	0	15	38.9±19.4
K	3	4	5	6	2	1	1	0	0	1	ND
M	4	3	5	6	2	9	0	9	8	1	25.8±13.1
P	4	4	5	7	2	18	18	0	0	18	24.2±14.5
S	5	3	5	6	2	7	1	6	5	2	28.7±16.8
U**	5	4	5	7	2	34	34	0	0	34	39.4±17.2
V	6	3	5	6	2	5	0	5	5	0	26.4±11.6
X	6	4	5	7	2	21	21	0	0	21	28.1±11.3
Y	7	4	5	6	2	1	1	0	0	1	ND
Z	7	4	5	7	2	5	5	0	0	5	29.6±21.4
n.c.^c^	2	4	5	7	3	2	2	0	0	2	ND
n.c.	3	3	5	7	2	1	1	0	0	1	ND
n.c.	3	4	5	7	1	1	1	0	0	1	ND
n.c.	6	4	4	7	2	1	1	0	0	1	ND
n.c.	6	4	5	6	2	1	0	1	1	0	ND
Total						136	111	25	22	114	32.4±16.1

^a^ PSI score (mean ± SD) of corresponding patients.

^b^ ND: not done.

^c^ Not classifiable using the scheme proposed by Degrange et al. (2009).

: J VS M p<0.001, J VS P p = 0.001, J VS S p = 0.008, J VS V p<0.001, J VS X p<0.001, J VS Z p = 0.016;

: U VS E p = 0.031, U VS M p<0.001, U VS P p<0.001, U VS S p<0.001, U VS V p<0.001, U VS X p<0.001, U VS Z p<0.001.

**Table 3:** Comparison of macrolide resistant rate among *M. pneumoniae* clinical isolates with different VNTRs.

Group	Number of repeats in Mpn1	Number of repeats in Mpn13-14-15-16	Isolates		χ^2^	P value
			Sensitive	Resistant		
1	2/3/4/5/6	3-5-6-2	21	4	97.5	<0.001^a^
2	2/3/4/5/6/7	4-5-7-2	0	103	10.9	0.072^b^
3	3	3-5-7-2	0	1	2.8	0.001^c^
	6	4-4-7-2	0	1		
	3/6/7	4-5-6-2	1	2		
	3	4-5-7-1	0	1		
	2	4-5-7-3	0	2		

^a^ group 1 VS group 2;

^b^ group 2 VS group 3;

^c^ group 3 VS group 1.

### Analysis on Clinical features of patients infected with different MLVA types

PSI scores of the patients had been calculated. As shown in table 2, comparing with types M (4-3-5-6-2), P (4-4-5-7-2), S (5-3-5-6-2), V (6-3-5-6-2), X (6-4-5-7-2) and Z (7-4-5-7-2), PSI scores were significantly higher in patients infected with *M. pneumoniae* types J (3-4-5-7-2) and U (5-4-5-7-2) (p<0.05 or p<0.001). Main clinical features of patients were compared between two groups (group 1: MLVA types “J & U” and group 2: other MLVA types), including age, comorbidities, smoking status, main symptoms, total duration of fever and cough, length of hospitalization (table 4). No differences had been found in gender, smoking status and main clinical symptoms. Patients in group 1 were significantly older than those of group 2 (p = 0.006), and had longer total duration of cough (p = 0.011).

**Table 4:** Comparison of Clinical features in CAP patients between two MLVA groups.

	Group 1^a^	Group 2^b^	p value
Patients number (n)	49	87	
Gender male, n (%)	24 (49.0, 35.0–63.0)	37 (42.5, 32.1–52.9)	0.479
Age (years)	35.6±14.6	29.3±11.5	0.006
Comorbidities, n (%)	4 (8.2, 0.5–15.9)	3 (3.4, −0.4–7.2)	0.429
Smoking, n (%)	9 (18.4, 7.6–29.2)	12 (13.8, 6.6–21.0)	0.622
Fever, n (%)	46 (93.9, 87.2–100.6)	84 (96.6, 92.8–100.4)	0.68
T_max_ (°C)	39.1±0.7	39.2±0.7	0.719
Cough, n (%)	49 (100.0, 100.0–100.0)	84 (96.6, 92.8–100.4)	0.303
Sputum, n (%)	37 (75.5, 63.5–87.5)	67 (77.0, 68.2–85.8)	1.000
WBC (×10^9^/L)	7.6±2.3	7.6±2.6	0.892
PSI score	39.3±17.7	28.5±13.8	<0.001
Total duration of fever (days)	5.8±3.7	5.2±3.0	0.288
Total duration of cough (days)	17.8 (10.0)	13.6 (7.0)	0.011
Length of hospitalization (days)	6 (14)	6 (10)	0.472

Note: The data are presented as n (%, 95% confidence intervals), mean ± SD or median (IQR).

^a^ Group 1: patients infected with MLVA types “J & U”;

^b^ Group 2: patients infected with other MLVA types.

## Discussion

*M. pneumoniae* is an important pathogen in adolescents and adults CAP patients, with a positive rate as 14.3% (136/954) by culture in our study. Compared with those without *M. pneumoniae* infection, patients with *M. pneumoniae* pneumonia have several specific clinical characteristics, including younger age, less comorbidities or smoker, more proportion of cough, and lower PSI scores, similar with the findings from CAPNETZ [24]. Based on clinical data of the defined patients group and MLVA genotyping method (with a good discriminatory ability of *M. pneumoniae*), we investigate the possible clinical relevance of different MLVA genotypes.

Using MLVA as an individual identification method, all of 136 *M. pneumoniae* clinical isolates were divided into 18 MLVA types, with U (5-4-5-7-2), X (6-4-5-7-2), P (4-4-5-7-2), J (3-4-5-7-2) and E (2-4-5-7-2) as the most prevalent. The distribution of MLVA types was consistent with two previous studies in China [9], [14], slightly different from the most common types, P (4-4-5-7-2), U (5-4-5-7-2), O (4-3-6-6-2), J (3-4-5-7-2) and E (2-4-5-7-2), reported by Degrange et al. [7], which might result from geographical differences.

All of the 114 macrolide-resistant isolates covered 15 of 18 MLVA types (table 2), which parallels previous findings from China and France [7], [9], [12], [14]. However, we found that 71.4% (5/7) of type S (5-3-5-6-2), 88.9% (8/9) of type M (4-3-5-6-2) and 100% (5/5) of type V (6-3-5-6-2) were not resistant to macrolides. Although these isolates only accounted for 15.6% (21/136) of all isolates, they occupied 81.8% (18/22) of all macrolide non-resistant isolates. The VNTR locus Mpn1 was reported to be unstable in both clinical isolates and in laboratory passages [8], [9], and the VNTR profiles of Mpn13-14-15-16 were all as 3-5-6-2, which suggested that MLVA types M (4-3-5-6-2), S (5-3-5-6-2) and V (6-3-5-6-2) might be the same type. Moreover, the rate of macrolide non-resistance of isolates harboring Mpn13-14-15-16 as 3-5-6-2 was significantly higher than those with other VNTRs profiles. Similarly, among 31 macrolide resistant isolates of *M. pneumoniae*, reported by three studies from France and the United States [7], [8], [13], there were only three isolates having Mpn13-14-15-16 profile as 3-5-6-2. Mpn 13 was located in intergenic region, and the other three were located in open reading frames, encoding hypothetical proteins [7]. Further studies on more non-resistant *M. pneumoniae* isolates are needed to determine whether there is possible association between Mpn13-14-15-16 profile of 3-5-6-2 and macrolide susceptibility.

Chalker et al. had found that patients with MLVA type M (4-3-5-6-2) did not all have the same symptoms or severity of infection [15]. However, limited by the small sample amount, they did not to investigate the association of particular types with clinical features. Likewise, Benitez did not analyze the possible clinical relevance of MLVA types [8]. Herein, we found that patients infected with *M. pneumoniae* MLVA types U (5-4-5-7-2) and J (3-4-5-7-2) might have higher morbidity. Again, MLVA type U (5-4-5-7-2) and J (3-4-5-7-2) might be the same type, considering the profiles of these two types differ only in Mpn1, the unstable locus [8]. This is the first observation of the clinical relevance of MLVA types.

There are two limitations in this study. First, since we have used *M. pneumoniae* cultures for both MLVA typing and determination of antibiotic resistance, and since cultures have much lower sensitivity in identifying *M. pneumoniae* in clinical samples we might have a bias towards patients with higher bacterial burden. In most recent papers, the clinical MLVA typing was performed directly on throat swabs without culturing [16]. Another limitation is that no children have been enrolled in our study. We could not answer whether there is any specific MLVA type responsible for the higher prevalence of *M. pneumoniae* in children than adolescents and adults [1]. But the distribution pattern of MLVA types we found here was similar with the finding from a study on pediatrics [9].

In conclusions, macrolide-resistance was not commonly observed in *M. pneumoniae* harboring Mpn13-14-15-16 profile as 3-5-6-2. Compared to those infected with the other types, CAP patients infected with *M. pneumoniae* types U (5-4-5-7-2) and J (3-4-5-7-2) might have higher clinical severity.

## Supporting Information

Table S1 **In vitro activity of nine antimicrobials against 136** ***M. pneumoniae*** **isolates.**
(DOCX)
