# Supplementary material for: Specific Multilocus Variable-Number Tandem-Repeat Analysis Genotypes of Mycoplasma pneumoniae Are Associated with Diseases Severity and Macrolide Susceptibility
Source: PLoS One. 2013 Dec 18;8(12):e82174. doi: 10.1371/journal.pone.0082174 (PMC3867324; doi:10.1371/journal.pone.0082174)
Supplement: Table S1 — In vitro activity of nine antimicrobials against 136 M. pneumoniae isolates. (DOCX) [file pone.0082174.s001.docx]

Table S1. In vitro activity of nine antimicrobials against 136 *M. pneumoniae* isolates.

|  |  | Total isolates | |  | MPFH |
| --- | --- | --- | --- | --- | --- |
|  |  | MIC_50_ | MIC_90_ |  | MIC |
| Erythromycin |  | 256 | >256 |  | ≤0.004 |
| Clarithromycin |  | 256 | >256 |  | ≤0.004 |
| Azithromycin |  | 64 | 128 |  | ≤0.004 |
| Tetracycline |  | 0.25 | 0.5 |  | 0.25 |
| Minocycline |  | 0.25 | 0.5 |  | 0.125 |
| Levofloxacin |  | 0.5 | 0.5 |  | 0.25 |
| Ciprofloxacin |  | 0.5 | 1 |  | 0.5 |
| Gatifloxacin |  | 0.064 | 0.064 |  | 0.064 |
| Moxifloxacin |  | 0.064 | 0.064 |  | 0.032 |
